# Risk factors for target vessel endoleaks after physician-modified fenestrated or branched endovascular aortic arch repair: A retrospective study

**DOI:** 10.3389/fcvm.2023.1058440

**Published:** 2023-03-21

**Authors:** Zhipeng Chen, Dongsheng Fu, Cheng Liu, Yi Jin, Chaohui Pan, Subinur Mamateli, Xiaochen Lv, Tong Qiao, Zhao Liu

**Affiliations:** Department of Vascular Surgery, Affiliated Drum Tower Hospital, Medical School of Nanjing University, Nanjing, China

**Keywords:** fenestrated or branched endovascular aortic arch repair, target vessel-related endoleak, aortic dissection, thoracic aortic aneurysm, risk factors

## Abstract

**Objective:**

Fenestrated or branched endovascular aortic arch repair (fb-arch repair) is an effective option for treating complex aortic arch lesions, including thoracic aortic aneurysms and aortic dissections. However, the relatively high rate of re-intervention due to target vessel (TV)-related endoleaks have raised concerns. This study aimed to determine risk factors for TV-related endoleaks after fb-arch repair.

**Methods:**

This was a retrospective analysis of all patients undergoing fb-arch repair between 2017 and 2021in nanjing drum tower hospital of China. All the patients underwent computed tomography angiography (CTA) before surgery; at discharge; and at 3 months, 6 months, and yearly post-discharge. All procedures are performed with physician modified grafts. Two experienced vascular surgeons used CTA and vascular angiography data to assess endoleaks. The study endpoints were mortality, aneurysm rupture, and emergence of and re-intervention for TV-related endoleaks.

**Results:**

During the follow-up period, 218 patients underwent fb-arch repair. There were seven perioperative deaths and four deaths during follow-up (two myocardial infarctions and two malignancies). There were nine additional patients who were excluded from the study (two strokes, three with abnormal aortic arch anatomy, and four with insufficient clinical data). Among the 198 patients considered (mean age, 59 ± 13.3 years; 85% male), 309 branch arteries were revascularized. A total of 35 TV-related endoleaks were identified in 28 patients during a mean follow-up of 23 ± 14 months (median 23, IQR 26.3): six type Ic, 4 type IIIb, and 20 type IIIc endoleaks. Patients in the endoleak group had greater aortic arch segment diameters (43.1 ± 5.1 vs. 40.3 ± 4.7; *P *= 0.004) and a greater number of TVs revascularized (2.0 ± 0.8 vs. 1.5 ± 0.8; *P *= 0.004) than those in the non-endoleak group. However, the morphological classification of the aortic arch did not seem to affect the occurrence of TV endoleaks (13%, 14%, and 15% for type І, II, and III aortic arches, respectively; *P *= 0.957). Pre-sewing branch stents in the fenestration position reduced the risk of TV endoleaks (5% vs. 14%; *P *= 0.037). Additionally, in TVs affected by aortic aneurysm or dissection, the risk of endoleaks increased after reconstruction (17% vs. 8%; *P *= 0.018). The incidence of secondary TV-related endoleaks after fb-arch repair was 14.1%.

**Conclusion:**

The data from this study showed that the incidence of secondary target vessel related endoleaks after fb-arch repair is approximately 14.1%. Additionally, patients with a larger aortic arch diameter or more revascularized arteries during surgery were at increased risk TV-related endoleaks. The target vessels originating from the false lumen or aneurysm sac are more prone to endoleaks after reconstruction. Finally, prefabricated branch stents reduced risk of TV-related endoleaks.

## 1. Introduction

Aortic arch pathologies involving the supra-aortic vessels are a major challenge for surgeons. Traditional open or hybrid surgeries are highly traumatic for patients. Despite surgical modifications and improved postoperative care, open or hybrid surgeries continue to have relatively high rates of morbidity and mortality (1, 2). Total endovascular aortic arch repair is a feasible approach for managing complicated aortic arch diseases (3, 4); however, some concern regarding the associated high re-intervention rate remains. Owing to Chinese policy restrictions, the development and promotion of company-manufactured devices (CMDs) in China has lagged behind those in other countries. Most fenestrated or branched endovascular aortic arch repair (fb-arch repair) procedures use physician-modified endografts (PMEGs) rather than CMDs. Notably, PMEG use may result in higher rates of complications and re-intervention events than CMD use due to inconsistencies in stent graft modification standards (5).

Endoleaks are the primary cause of re-intervention after fenestrated or branched endovascular aortic repair (F/BEVAR), and target vessel (TV)-related endoleaks occur more frequently than those around the main stent graft. TV-related endoleaks are common, as F/BEVAR is common in those with complex aortic diseases and modular endografts. The following are the three main types of endoleaks associated with the TVs: type Ic, which is caused by retrograde blood flow from the distal end of a bridging stent; type IIIb, which involves a tear or break in the fabric of the bridging stent; and type IIIc, which is defined as poor connections between the bridging stent and fenestration ring, directional branch, or mini-cuffs. Among primary and secondary endoleaks, type IIIc is the most predominant TV-related endoleak type, accounting for 85% and 55%, respectively (6). Progression to type I and type III endoleaks often leads to aneurysm sac enlargement, thereby increasing the risk of aneurysm rupture. The latest guidelines recommend the treatment of type I and type III endoleaks (7, 8). The incidence of TV-related endoleaks in the visceral segment after F/BEVAR for thoracoabdominal aortic aneurysms ranges from 16.4% to 35.7% (9, 10). The development of fb-arch repair occurs slightly later than that of F/BEVAR in the visceral area, and there are only few studies on TV-related endoleaks after fb-arch repair.

This study aimed to examine the incidence of secondary TV-related endoleaks among patients who underwent fb-arch repair and to identify risk factors for secondary TV-related endoleaks using patient data from a high-volume, single center in China.

## 2. Materials and methods

### 2.1. Study population

This retrospective study was approved by the Ethics Committee of Nanjing Drum Tower Hospital (registration number: 2017-015-05), and all patients provided consent for their participation. At our hospital, 218 patients underwent fb-arch repair for 62 thoracic aortic aneurysms, 137 chronic dissections, 10 intramural hematomas, and 9 penetrating aortic ulcers involving the arch between June 2017 and September 2021. All of the patients received elective operations. 20 patients were excluded from the study due to deaths unrelated to endoleaks and other reasons. Aortic imaging data of the remaining 198 patients were obtained *via* computed tomography angiography (CTA). All patients underwent CTA examination before surgery; before discharge; 3, 6, and 12 months after discharge; and every year thereafter. Some patients (*n* = 63) sought an immediate CTA examination due to symptoms; therefore, their follow-up schedules were adjusted accordingly. All CTA images or angiographic data were reviewed by vascular radiologists and senior vascular surgeons.

### 2.2. Study design

Custom-manufactured devices (CMDs) are not widely used in mainland China due to health insurance policy requirements and cargo delivery time issues. PMEGs, which have been developed rapidly and widely in China, were used to treat all included patients. 3D printing, which may be used to improve the accuracy of fenestration (11), was used in this study to facilitate the treatment of patients with ≥2 TVs requiring reconstruction. Indications for surgery included thoracic aortic aneurysms or aortic dissection with rupture, chest or back pain, an asymptomatic aneurysm sac diameter of >5.5 cm or diameter increased by >5 mm within 6 months, intramural hematoma with a thickness of greater than 10 mm or persistent increase in size, combined with pleural effusion, or penetrating aortic ulcers >5 mm in depth with an insufficient landing zone due to pathologies involving supra-aortic vessels. Based on the patient's anatomy and lesion characteristics, we reconstructed branch vessels in one of the following three ways: *in situ* fenestration, on-site fenestration, or pre-sewn cuffs or branches.

All *in situ* fenestrations were performed using a combination of a steerable sheath and a needle to rupture the membrane. The diameter of the area of each fenestration was adjusted according to the size of the branch vessel opening, and a nitinol ring was used to mark the area around which sutures were required. The following bridging stents were implanted within each fenestration area: bridging stents, including Viabahn (W. L. Gore, Flagstaff, AZ); Fluency (BD/Bard, Murray Hill, NJ, United States), and the iliac branch of the endovascular aortic repair graft. In the early stages of this study, some bridging stents were relined using Omnilink (Abbott Vascular, Santa Clara, CA, United States) to prevent kinking. The mini-cuff included a 3–5-mm Viabahn, which was shortened and anastomosed end-to-side to the main stent graft using 5-0 Ethibond sutures (Ethicon, West Somerville, NJ). The directional branch was sutured using 1–1.5-cm Viabahn. The largest outer diameter of the entire aortic arch and the outer diameter of the aorta at the level of the branch opening were referred to as D1 and D2, respectively.

### 2.3. Procedure

All operations were performed under general anesthesia in a hybrid operating room equipped with a fixed fluoroscopy C-arm. PMEGs were performed using Valiant (Medtronic, Minneapolis, MN, USA; *n* = 57), Ankura (Lifetech Scientific, Shenzhen, China; *n* = 123), or Zenith (Cook, Bloomington, IN, USA; *n* = 18) platforms. Access was obtained through femoral and left brachial arteries, with the left common carotid and right subclavian arteries selected when necessary. Fenestrations or inner branches were preferred in cases in which TVs originated from the true lumen, and fenestrations were close to TV orifices. Mini-cuffs or outer branches were preferred if the TV originated from the false lumen and if there was sufficient distance between the main stent graft and the TV orifices. Steerable sheaths, such as FuStar (Lifetech Scientific Inc., Shenzhen, China), were used to facilitate TV cannulation and dissection flap puncture. Temporary diameter-reducing ties were used in all patients to ensure rotational and axial movement of the main stent graft, thus facilitating TV catheterization. After TVs were positioned and bridging stents were ready for deployment, reducing ties were removed, allowing for free movement of the main stent graft. Balloon molding of bridging stents is essential for eliminating gaps and preventing primary endoleaks. Anticoagulants were not routinely used after surgery, but we recommend that patients be treated with dual antiplatelet therapy (aspirin + clopidogrel) for 1–3 months after surgery, followed by a change to a single antiplatelet agent and adjustment of the dosing regimen based on follow-up results, according to requirements (12).

### 2.4. Statistical analysis

Categorical variables are expressed as numbers and percentages, and continuous variables are expressed as mean ± standard deviation or median, as appropriate. The Pearson chi-squared test was used to compare nominal data. Further, the student's *t*-test and Mann-Whitney *U*-test were used to compare mean values. Univariate and multivariate analyses were performed to identify risk factors for TV-related endoleaks among anatomic and stent graft-related variables. Odds ratios (OR) with 95% confidence intervals (CI) were used to reflect the odds of an event. Values of *P* < 0.05 were considered statistically significant. All statistical analyses were performed using SPSS version 25.0 software (IBM Corp., Armonk, NY, United States).

## 3. Results

### 3.1. Baseline patient characteristics and demographics

**Figure 1:**
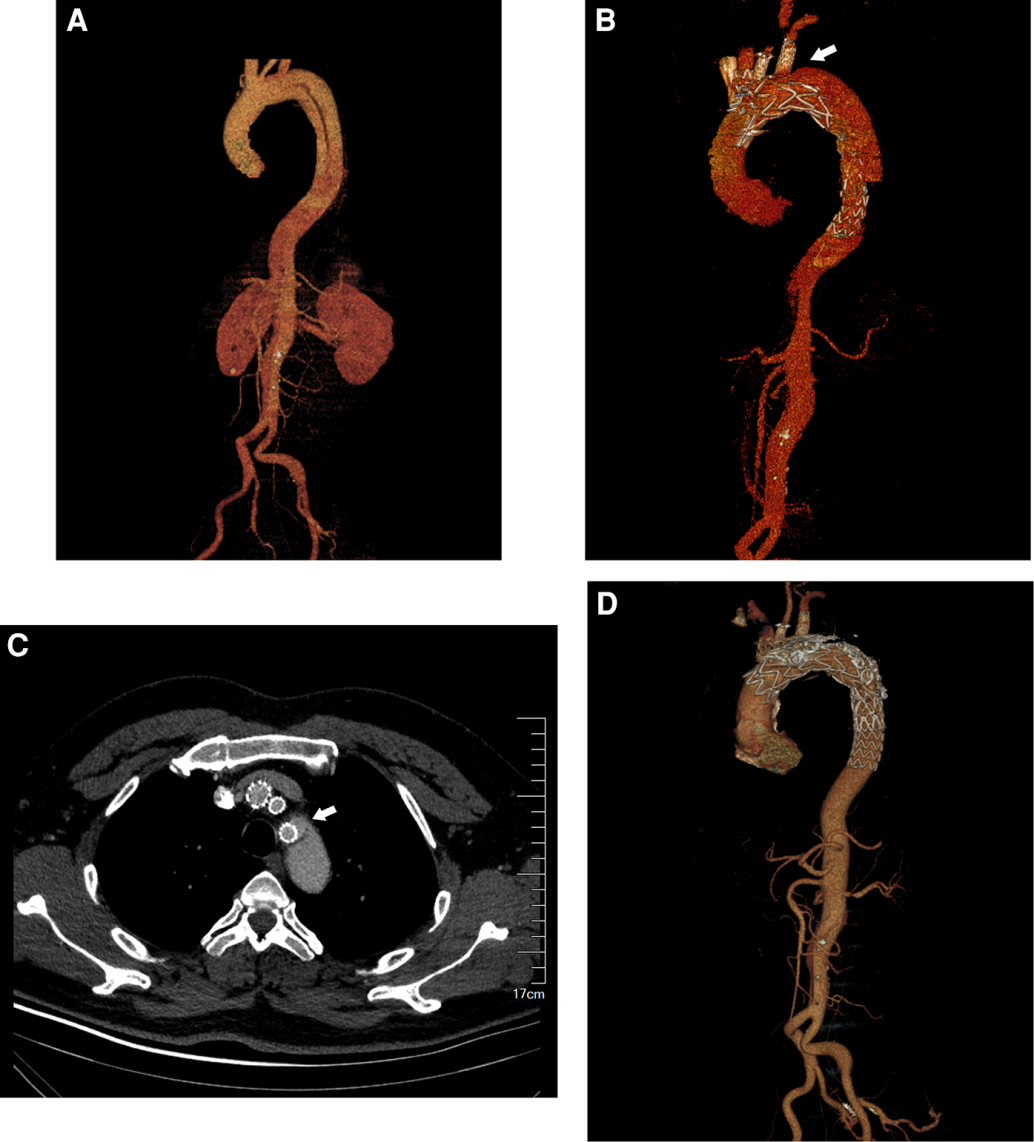
A 45-year-old male patient underwent fb-arch repair for chronic aortic dissection. The patient developed type IIIc endoleak around the left subclavian artery during the follow-up. (**A**) The CTA imaging of the aortic arch involved by dissection. (**B**) The CTA imaging of the Type IIIc endoleak near the left subclavian artery after fb-arch repair. (arrow). (**C**) The cross-sectional image of the Type IIIc endoleak (arrow). (**D**). After two false lumen coil embolization, the original endoleak disappeared and the aorta was well remodeled.

A total of 218 patients underwent fb-arch repair at our center throughout the study period: 62 with thoracic aortic aneurysms, 137 with chronic dissections, 10 with intramural hematomas, and 9 with penetrating aortic ulcers involving the arch. Perioperative deaths occurred in seven patients: five with retrograde type A aortic dissections, one with thoracic aortic rupture, and one with myocardial infarction. There were four deaths during follow-up (two myocardial infarctions and two malignancies). In addition, nine patients were excluded from the study (two experienced strokes, three had abnormal aortic arch anatomy, and four had insufficient clinical data). We estimated 30-day and 24-month survival rates as 96.8% and 95.4%, respectively. Among the remaining 198 patients (mean age, 59 ± 13.3 years; 85% male) with aortic arch diseases, 309 branch arteries were revascularized using 172 fenestrations and 137 inner- or outer-branch stents.A total of 35 TV-related endoleaks were identified among 28 patients during a mean follow-up period of 23 ± 14 months (median 23, IQR 26.3): six patients with type Ic (retrograde from the distal end of the branch), four with type IIIb (bridging stent fabric tear), and 20 with type IIIc endoleaks (detached stent or loose bridging stent connection). As depicted in Figure 1, A 45-year-old male patient developed type IIIc endoleak around the left subclavian artery during the follow-up. After two false lumen coil embolization, the endoleak disappeared and the aorta was well remodeled. No significant differences were observed in patient demographics or the prevalence of comorbidities between patients with and without a TV endoleak (Supplementary Table S1). The characteristics of patients and TVs were explored to identify risk factors for TV-related endoleaks after fb-arch repair.

### 3.2. Risk factors for TV-related endoleaks after fb-arch repair

No significant between-group differences were noted in terms of body mass index and length of hospital stay post-procedure. However, the number of revascularized TVs per individual appeared to affect the risk of TV-related endoleak. Patients in the endoleak group had a greater number of reconstructed vessels than those of the non-endoleak group (Table 1, 2.0 ± 0.8 vs. 1.5 ± 0.8; *P* = 0.004). Univariate logistic regression analysis revealed that TVs per patient and ≥2 TVs were potential risk factors for endoleaks; however, considering the problem of collinearity, we chose ≥2 TVs for the final multivariate logistic regression. Subsequently, ≥2 TVs was identified as an independent risk factor for TV-related endoleaks after fb-arch repair (Table 2: OR, 3.849; 95% CI, 1.633–9.075; *P* = 0.002).

**Table 1:** Factors with the potential to affect TV-related endoleak occurrence after fb-arch repair.

	All patients (*N* = 198)	Target vessel endoleaks (*N* = 28)	No target vessel endoleaks (*N* = 170)	*P*
BA diameter, mm	14.3 ± 1.9	14.6 ± 1.9	14.3 ± 1.8	0.411
LCCA diameter, mm	9.2 ± 1.6	9.5 ± 1.4	9.1 ± 1.6	0.213
LSA diameter, mm	11.3 ± 1.7	11.6 ± 1.6	11.3 ± 1.7	0.416
D_1_, mm	40.7 ± 4.8	43.1 ± 5.1	40.3 ± 4.7	**0.004**
BMI	25.6 ± 3.4	26 ± 3.4	25.5 ± 3.4	0.538
Hospital LOS after procedure, d	12.8 ± 4.5	13.2 ± 4.5	12.7 ± 4.6	0.580
Procedure time, min	267.1 ± 79.7	296.7 ± 85.8	262.3 ± 77.9	**0**.**034**
Target vessels per patient	1.6 ± 0.8	2.0 ± 0.8	1.5 ± 0.8	**0**.**004**
≥ 2 target vessels	72 (36)	18 (64)	54 (32)	**0**.**001**
Get NOAC therapy	34 (17)	6 (21)	28 (16)	0.649
Aortic dissection	130 (66)	22 (79)	108 (64)	0.120
Morphological classification of the aortic arch				0.957
Type I	53	7/53 (13)	46	
Type II	106	15/106 (14)	91	
Type III	39	6/39 (15)	33	
Brand of the main graft				0.477
Lifetech	123	15/123 (12)	108	
Meditronic	57	9/57 (16)	48	
Cook	18	4/18 (22)	14	

BA, brachiocephalic artery; LCCA, left common carotid artery; LSA, left subclavian artery; D_1_: maximum diameter of the aortic arch; BMI, body mass index; LOS, length of stay; DOAC, direct oral anticoagulants.

All data are presented as number (%) or mean ± standard deviation.

**Table 2:** Univariate and multivariate analysis of risk factors for TV-related endoleaks in 198 patients who had previously undergone fb-arch repair.

Variables	Univariate analysis		Multivariate analysis	
	OR (95% CI)	*P*	OR (95% CI)	*P*
D_1_, mm	1.128 (1.044–8.986)	**0** **.** **042**	1.130 (1.033–1.236)	**0**.**008**
Target vessels per patient	1.924 (1.214–3.050)	**0**.**005**	−	** **
≥ 2 target vessels	3.867 (1.673–8.936)	**0**.**002**	3.849 (1.633–9.075)	**0**.**002**

**Figure 2:**
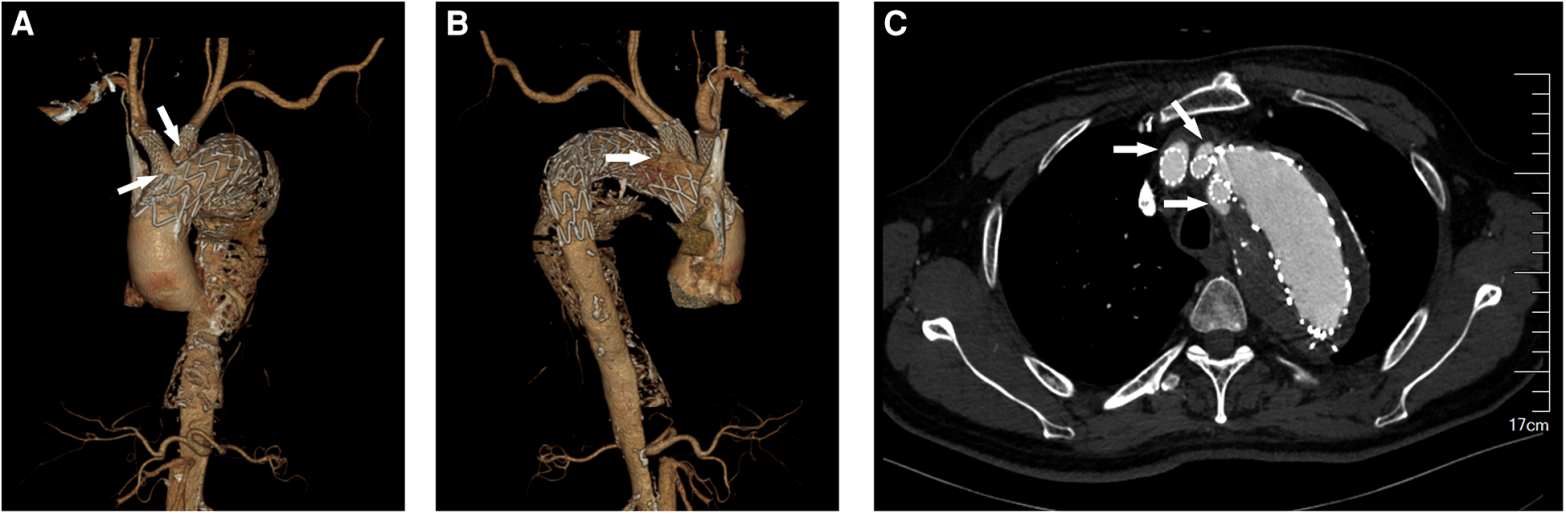
A 69-year-old male patient underwent fb-arch repair for a postdissection aortic arch aneurysm. Probably because of the large aortic arch diameter, there seems to be endoleaks around the reconstructed supra-aortic vessels during the follow-up. (**A**) The CTA imaging of the Type IIIc endoleaks near the brachiocephalic trunk and the left common carotid artery after fb-arch repair. (arrow). (**B**) The CTA imaging of the Type IIIc endoleak near the left subclavian artery after fb-arch repair. (arrow). (**C**) The cross-sectional image of the Type IIIc endoleaks (arrow).

Patients in the endoleak group had a greater aortic arch diameter than those in the non-endoleak group (D_1_: 43.1 ± 5.1 vs. 40.3 ± 4.7; *P* = 0.004). The use of D_1_ as a condition in univariate and multivariate analyses revealed that D_1_ was another independent risk factor for TV-related endoleaks (Table 2: OR, 1.130; 95% CI, 1.033–1.236; *P* = 0.008). During the follow up, we found a patient with the maximum aortic diameter higher than 65 mm had TV-related endoleaks around all three supra-aortic vessels after fb-arch repair (Figure 2). The operation time in the endoleak group was longer than that in the non-endoleak group (296.7 ± 85.8 vs. 262.3 ± 77.9; *P* = 0.034). This finding may be affected by the need for reconstruction of a greater number of branches in patients of the endoleak group.

Postoperative treatment with direct oral anticoagulants and anatomic aortic arch type were not found to affect the occurrence of TV-related endoleak. TV-related endoleaks were observed in 17% of patients with aortic dissection after fb-arch repair compared to 9% of those with other diseases involving the aortic arch (*P* = 0.120). The main grafts included Lifetech products from China and other brands from overseas. Although the fabric and stent design of products differed, no significant between-group differences were noted in the incidence of TV-related endoleaks among those with different graft products (12%, 16% and 22%, *P* = 0.477; Table 2).

### 3.3. Risk factors for TV-related endoleaks among 309 TVs after fb-arch repair

Endoleak-related risk factors were identified among 309 TVs. First, the effect of revascularization method on the occurrence of endoleak was assessed, revealing no significant between-group differences in the incidence of TV-related endoleaks. The revascularization methods that were considered included *in situ* fenestration (10 endoleaks among 72 TVs, 14%), on-site fenestration (20 endoleaks among 146 TVs, 14%), and pre-sewn cuffs or branches (5 endoleaks among 91 TVs, 5%; *P* = 0.112; Table 3). However, TVs revascularized with pre-sewn cuffs or branches appeared to result in a lower rate of endoleaks than those revascularized *via* other methods (5% vs. 14%, *P* = 0.037; Table 3). Similar results were observed when the effects of different bridging stent type use were assessed, revealing that the risk of endoleaks in TVs reconstructed with Fluency was slightly higher than that in Viabahn (13% vs. 8%, *p* = 0.249; Table 3). Importantly, the occurrence of TV-related endoleaks after surgery depended on whether TVs are affected by aortic arch lesions. The occurrence of TV-related endoleaks in branch vessels affected by aortic arch pathologies was 17%, which was higher than that in branch vessels not affected by pathologies (8%, *P* = 0.018, Table 3). In the multivariate analysis of endoleak-related risk factors among TVs, we determined that non-pre-sewn cuffs or branches (OR, 2.951; 95% CI, 1.091–7.980; *P* = 0.033), TVs affected by pathologies (OR, 2.107; 95% CI, 1.015–4.372; *P* = 0.045), and aortic diameter at the level of the TV opening (D_2_) (OR, 1.059; 95% CI, 1.002–1.120; *P* = 0.043) were independent risk factors for TV-related endoleaks (Table 4).

**Table 3:** Potential influencing factors for target vessel-related endoleaks among 309 target vessels after fb-arch repair.

	All target vessels (*N* = 309)	Target vessel endoleaks (*N* = 35)	No target vessel endoleaks (*N* = 274)	*P*
Endoleak position				0.954
BA	44	5/44 (11)	39	
LCCA	77	8/77 (10)	69	
LSA	188	22/188 (12)	166	
Modification method				0.112
*In situ* fenestration	72	10/72 (14)	62	0.433^a^
On-site fenestration	146	20/146 (14)	126	
Pre-sewn cuffs or branches	91	5/91 (5)	86	**0**.**037**^b^
Brand of bridging stent				0.473
Bard Fluency	164	21/164 (13)	143	0.249^c^
Gore Viabahn	108	9/108 (8)	99	
Iliac branch of EVAR graft	37	5/37 (14)	32	
TVs affected by pathologies	104 (34)	18 (51)	86 (31)	**0**.**018**
D_2_, mm	36.8 ± 6.5	39.1 ± 6.5	36.5 ± 6.4	**0**.**023**
Oversize ratio	1.105 ± 0.049	1.099 ± 0.052	1.106 ± 0.048	0.427
Bridge stent diameter, mm	10.5 ± 1.5	10.8 ± 1.7	10.5 ± 1.5	0.220
Bridge stent length, mm	45.5 ± 10.4	45.1 ± 11.1	45.6 ± 10.3	0.821

BA, brachiocephalic artery; LCCA, left common carotid artery; LSA, left subclavian artery; D_1_: maximum diameter of the aortic arch; TV, target vessel.

^a^ *In situ* fenestration compared with the other two methods.

^b^ Pre-sewn cuffs or branches compared with the other two methods.

^c^ Bard Fluency compared with Gore Viabahn; D_2_, aortic diameter at the level of TV orientation.

All data are presented as number (%) or mean ± standard deviation.

**Table 4:** Univariate and multivariate analyses of risk factors for target vessel (TV)-related endoleaks among 309 TVs after fb-arch repair.

Variables	Univariate analysis		Multivariate analysis	
	OR (95% CI)	*P*	OR (95% CI)	*P*
Not pre-sewn cuffs or branches	2.745 (1.030–7.317)	**0.044**	2.951 (1.091–7.980)	**0**.**033**
TVs affected by pathologies	2.315 (1.138–4.709)	**0**.**021**	2.107 (1.015–4.372)	**0**.**045**
D_2_, mm	1.064 (1.008–1.123)	**0**.**025**	1.059 (1.002–1.120)	**0**.**043**

## 4. Discussion

Total endovascular repair using fenestrated and branched technology is an appropriate option for the treatment of aortic arch aneurysms and chronic aortic dissection involving the supra-aortic vessels. Although total endovascular treatment avoids damage caused by thoracotomy and circulatory arrest, F/BEVAR remains challenging for vascular surgeons. Marek et al. reported an 85% technical success rate for F/BEVAR, with 30- and 90-day mortality rates of 7% and 15%, respectively (10). These values were higher than those of the current study. Our data more closely mirrored the findings of a multicenter study from China, which estimated 30-day and 24-month survival rates as 97.5% and 94.9%, respectively (13). This was likely due to the high proportion of single fenestrations encountered in our study. Complete interruption of the false lumen or aneurysm sac perfusion greatly affects the prognosis of patients with aortic disease. In fact, in 62.7% of type B aortic dissections, an increase in aortic diameter 5-years after TEVAR was observed (14). Further, when the diameter of a dissection aneurysm exceeded 60 mm, risk of aneurysm rupture within one year reached 30% (15). Therefore, it is important to ensure that the stent graft fully covers the lesion area and actively correct large-flow endoleaks.

Owing to the modular design of the endograft used in the F/BEVAR procedure, TV-related endoleaks were the most common type of endoleak after surgery and were the most common cause of postoperative re-intervention. Kitagawa et al. (16) examined 30 patients with post-dissection TAAAs; despite remarkably good perioperative outcomes, up to 40% of patients underwent re-intervention for various endoleaks. The study further revealed that aortic diameter was closely associated with the incidence of TV-related endoleaks after F/BEVAR, with aortic arch diameters (D_1_) of the 28 patients with TV-related endoleaks significantly greater than those of the 170 patients without endoleaks. Similar results were observed when the occurrence of TV-related endoleaks after F/BEVAR in degenerative aneurysms was assessed (6). This finding may be explained by the fact that an enlarged aneurysm sac often increases the distance between fenestrations and TV orientation, both of which provide space for blood flow to enter the sac lumen due to a connection gap.

The present study revealed that patients with a greater number of TVs were more likely to have endoleaks. Patients with many TVs require revascularization, usually because the dissection entry point or aneurysm sac is close in proximity to the opening of visceral branch vessels. However, the presence of an increased quantity of TVs correspondingly increases the risk of accumulation. This study suggests that TVs revascularized using pre-sewn cuffs or branches are less likely to have endoleaks than those revascularized using *in situ* fenestration or on-site fenestration. This reduced risk of TV-related endoleaks may be associated with the fact that PMEGs are limited by struts during main stent modification, especially during F/BEVAR during which the diameter of the fenestration can exceed 1 cm. In particular, creation of a standard circle fenestration is difficult, and the connection with the branch stent is very short. In contrast, the connection between the mini-cuff or branch to the bridging stent when using in a standard round stent (usually Viabahn) is 3–15 mm.

Although no iCAST-covered stent, a widely used bridging stent, is available, many alternatives such as self-expanding covered stents (Fluency, Viabahn), balloon-expanding covered stents (Lifestream, BARD), and balloon-expanding bare mental stents (Omnlink, Abbott) can be used in China. A previous study showed that the probability of endoleak occurrence in those with balloon-expandable stents may be higher than that in those with self-expanding stent grafts; however, due to the limited number of cases considered, this study failed to support such prior conclusions. The present study suggested that TVs revascularized with Fluency stents appear to be more prone to endoleaks than those revascularized with Viabahn, a finding that may be due to the stiffness of Fluency stents. It is not uncommon for bridging stents to migrate or slip, mainly because mismatch between calibers of the bridging stent and fenestrations or branches may occur (17). The diameter of the bridging stent should be smaller than that of the branch vessel; therefore, the proximal end of the bridging stent should not enter the main graft to a great extent. TVs involved in pathologies are independent risk factors for TV-related endoleaks. The probability of entry into the false lumen around the opening of branch vessels is high, resulting in a certain distance between the actual opening of branch vessels and feneatrations, leaving room for endoleak blood flow to enter the false lumen. However, entry may occur at the distal end of a branch vessel. Alternatively, stent-induced entry may occur due to bridging stent implantation.

This study has some limitations. First, the sample size was insufficient for identifying risk factors for endoleaks after surgery with confidence. A larger number of positive samples would provide more convincing findings. Moreover, since an insufficient number of cases were considered, we were unable to perform a subgroup analysis of different types of TV-related endoleaks. There was a certain proportion of patients who were lost follow-up. Finally, all patients received PMEGs; therefore, findings may not be applicable in patients receiving CMDs.

In conclusion, fb-arch repair is an effective means for treating aortic arch pathologies; however, the relatively high incidence of TV-related endoleaks is concerning. Increased aortic arch diameter, TVs affected by aortic arch lesions, and the number of revascularized branches are independent risk factors for TV-related endoleaks after fb-arch repair. However, pre-sewn cuffs and branches were determined to be protective factors.

## Data Availability

The raw data supporting the conclusions of this article will be made available by the authors, without undue reservation.
