# Supplementary material for: Risk factors for target vessel endoleaks after physician-modified fenestrated or branched endovascular aortic arch repair: A retrospective study
Source: Front Cardiovasc Med. 2023 Mar 21;10:1058440. doi: 10.3389/fcvm.2023.1058440 (PMC10070968; doi:10.3389/fcvm.2023.1058440)
Supplement: Supplementary file 1 [file Table2.docx]

**Supplementary Table 1**. Demographic characteristics and comorbidities of 198 patients undergoing F/BEVAR with or without TV-related endoleaks

|  | All patients  (N = 198) | Target vessel endoleaks  (N = 28) | No target vessel endoleaks  (N = 170) | P |
| --- | --- | --- | --- | --- |
| Age, years | 59 ± 13 | 56 ± 11 | 60 ± 14 | 0.18 |
| Sex, male | 169 (85) | 25 (89) | 144 (85) | 0.73 |
| Cigarette smoking | 134 (68) | 20 (71) | 114 (67) | 0.65 |
| Alcohol consumption | 139 (70) | 21 (75) | 118 (69) | 0.55 |
| Hypertension | 183 (92) | 27 (96) | 156 (92) | 0.70 |
| Diabetes | 68 (34) | 10 (36) | 58 (34) | 0.87 |
| CKD | 45 (23) | 7 (25) | 38 (22) | 0.76 |
| COPD | 37 (19) | 5 (18) | 32 (19) | 0.90 |
| Stroke | 36 (18) | 6 (21) | 30 (18) | 0.63 |
| Coronary artery disease | 39 (20) | 5 (18) | 34 (20) | 0.79 |

All data except age (presented as mean ± standard deviation) are presented as number (%)
